# Tick-Borne Encephalitis Presenting as Brachial Plexus Injury: A Case Report

**DOI:** 10.1155/crdi/7003058

**Published:** 2025-02-22

**Authors:** Angelika Maksimiuk, Dominik Wawrzuta, Joanna Zajkowska

**Affiliations:** ^1^Medical Clinic “Wa-Med”, Wasilków 16-010, Poland; ^2^Department of Radiation Oncology, Maria Skłodowska-Curie National Research Institute of Oncology, Warsaw 02-034, Poland; ^3^Department of Infectious Diseases and Neuroinfections, Medical University in Białystok, Białystok 15-089, Poland

**Keywords:** neurologic deficit, neurological complications, tick-borne encephalitis

## Abstract

Tick-borne encephalitis (TBE) is a viral infection with variable clinical presentations, including neurological complications. We report a case of TBE in a 34-year-old farmer from an endemic region in Poland. The patient initially presented with paresis of the right brachial plexus. The diagnosis was challenging due to the absence of previous flu-like symptoms, often related to TBE infection. Neurological evaluations revealed paresis and muscle atrophy in the right upper limb, and serological tests confirmed TBE infection. Despite treatment efforts, neurological deficits persisted. This case highlights the need to consider TBE in the differential diagnosis of sudden-onset neurological disorders, especially in TBE-endemic regions, to ensure prompt intervention and prevent long-term complications.

## 1. Introduction

Tick-borne encephalitis (TBE) is an infectious disease caused by the TBE virus (TBEV) of the Flaviviridae family. This disease spreads primarily through tick bites, although infection can also occur by eating contaminated unpasteurized dairy products [1]. Some cases of TBE may remain asymptomatic and resolve independently, but more than 30% of patients exhibit symptoms [2, 3]. In most cases, TBE manifests itself as a two-phase disease. The first phase emerges after an incubation period of 4–10 days, characterized by flu-like symptoms such as fever, nausea, fatigue, headache, and vomiting. These symptoms typically subside within seven days. However, after a remission period of up to 7 days, more than half of the patients progress to the second phase, marked by high fever and neurological symptoms such as meningitis, encephalitis, myelitis, or radiculitis [4]. Some patients skip the initial flu-like phase, revealing only neurological sequelae. This atypical presentation often complicates the diagnostic process, leading patients to seek medical attention only when neurological complications arise. Even if we know that brachial plexus paresis is the most common neurological paresis caused by TBEV [5], published so far case reports focus on describing neurological disorders at the time of diagnosis, without describing their long-term course and complications [6].

## 2. Case Presentation

A 34-year-old farmer, previously diagnosed with bronchial asthma and residing in the Podlaskie Voivodeship, an endemic region of TBE in Poland, sought medical attention at the Neurological Department of Białystok Hospital due to suspected damage to the right brachial plexus. The patient first became concerned about his symptoms the day before admission, when he experienced numbness in the right upper limb, fever (up to 38°C), and loose stools that had started two days earlier. Upon detailed questioning, he revealed that similar symptoms had occurred previously but he had dismissed them, attributing them to physical exertion from his work and wildlife photography hobby in forests. Additionally, he had been experiencing malaise and headaches, for which he took nonsteroidal anti-inflammatory drugs (NSAIDs) about a month ago. The symptoms subsided following this treatment. He had not received TBE vaccination, denied consuming unpasteurized milk, and mentioned a tick bite from a year ago without undergoing antibiotic treatment.

Physical examination revealed paresis and muscle atrophy in the right upper extremity, with a positive Brudziński symphyseal sign, while other meningeal signs were negative. Computed tomography and magnetic resonance imaging revealed degenerative changes in the cervical spine and isolated pulmonary fibrosis. There was no evidence of nerve root compression that could explain the limb weakness. Furthermore, an electromyography (EMG) examination revealed degenerative changes within the right brachial plexus. Cerebrospinal fluid analysis revealed lymphocytic pleocytosis with a cell count of 24 lymphocytes/μL, while other parameters remained within normal limits (protein 44 mg/dL, chloride 118 mmol/L, and glucose 60 mg/dL). Serological blood tests confirmed the presence of positive IgM and IgG antibodies against the TBEV and positive IgG antibodies against *Borrelia burgdorferi*, as shown in Table 1. Serological or PCR tests of cerebrospinal fluid were not conducted.

The diagnosis of post-inflammatory damage to the upper segment of the brachial plexus resulting from infection was conclusively determined. The treatment regimen included rehabilitation for the right arm and intravenous therapy, which included ceftriaxone (2 g once daily) due to antibodies against *Borrelia burgdorferi*, and a combination of B vitamins. Despite treatment, there was only a modest improvement in the functionality of the right upper extremity. After 14 days, the patient opted to be discharged from the hospital, discontinuing further medical intervention.

**Figure 1:**
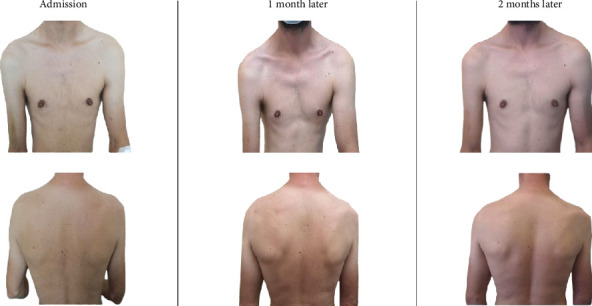
Progression of muscle atrophy of the right upper extremity.

The patient was discharged in stable condition, receiving instructions to continue outpatient physical therapy and attend scheduled follow-up appointments at the infectious disease clinic. Monthly follow-up visits indicated no new symptoms, and the right paresis gradually improved.  Figure 1 shows the progression of muscle atrophy in the right upper extremity during the follow-up visits. The patient was unable to fully raise his right arm, with atrophy noted in the pectoralis major and trapezius muscles on the right side. He returned for his final visit three months after admission and reported stable, ongoing paresis.

## 3. Discussion

Poland records over 300 cases of TBE annually, with the majority concentrated in the northeastern regions [7]. However, the entire country is at risk for TBE transmission [8]. At the patient's residence, in the Podlaskie Voivodeship, the morbidity rate of TBE in 2022 reached 11 cases per 100,000 population, marking the highest rate in the country [9]. TBE infections in Poland are predominantly attributed to tick bites, although cases resulting from consuming unpasteurized dairy products have also been documented, with the TBEV identified in 11% of cow milk samples [10].

**Figure 2:**
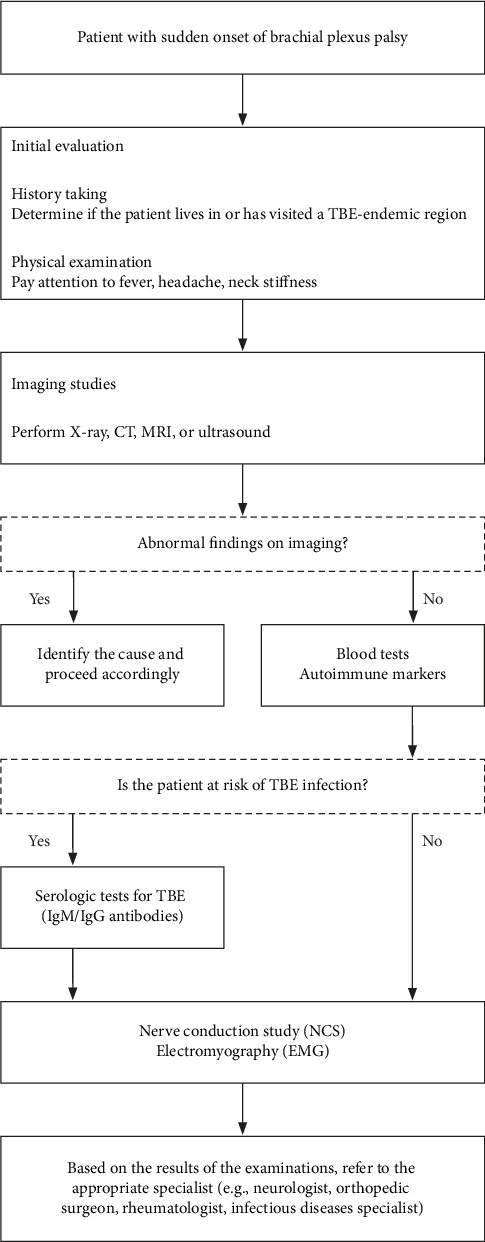
Diagnostic decision tree for sudden onset of brachial plexus palsy.

Most patients with tick-borne diseases may not be aware of a tick bite and may experience an asymptomatic course. Symptomatic individuals typically manifest general symptoms in the initial phase of the disease, including fatigue, fever, headaches, and muscle pain. Subsequently, up to 15% of patients progress to a second phase marked by the development of neurological symptoms, often accompanied by fever exceeding 40°C [2, 3]. The patient described in this case report denied experiencing the initial phase of the disease and reported the appearance of fever at the beginning of the second phase. The lack of symptoms could potentially be caused by taking NSAIDs. Given the diagnostic challenges associated with such atypical presentations, we have developed a diagnostic algorithm for brachial plexus palsy in the context of TBE infection risk, as illustrated in Figure 2. This algorithm is based on our institutional experience and aims to aid clinicians in the timely and accurate diagnosis of TBE.

The second phase involves inflammation of the central nervous system. Patients may experience various neurological symptoms associated with meningitis or encephalitis, such as ataxia, seizures, tremors, nerve palsies, or paresis. Analysis carried out in Poland revealed that 11% of patients with TBE experience enduring neurological sequelae, and 21% of them have brachial plexus paresis, reflecting the condition observed in the described patient [11]. It is crucial to consider serological testing for TBE in patients with lymphocytic meningitis or unexplained neurological deficits who reside in or have visited regions where TBE is prevalent. Although cases of plexus neuritis after TBE infection have been described in the literature, they primarily focus on short-term neurological problems [6]. Our extended follow-up demonstrated that these neurological problems are not temporary and can lead to long-term disturbances with muscle atrophy.

Currently, there is no specific antiviral treatment available for TBE. Typically, patients with TBE are hospitalized when they experience neurological complications. The standard of care involves providing supportive therapy based on the patient's symptoms [4]. This treatment approach may include antipyretics, analgesics, antiemetics, anticonvulsant medications, or drugs to reduce intracranial pressure [12]. The primary goal of treatment is to prevent both neurological and systemic complications and to minimize any additional damage to nerve cells.

## 4. Conclusions

This case report highlights the importance of considering TBE in the differential diagnosis of sudden-onset brachial plexus paresis, especially in endemic regions. Early recognition and serological testing for TBE are crucial for initiating appropriate treatment and management. Although there is no specific antiviral treatment for TBE, supportive care and rehabilitation can help mitigate the long-term neurological sequelae. However, chronic complications associated with this disease may not be recoverable and can lead to muscle atrophy.

## Figures and Tables

**Table 1:** Patient serological test results.

			Hospitalization (day 0)	End of follow-up (after 3 months)
Tick-borne encephalitis virus	IgM	Negative < 9 Doubtful 9–11 Positive > 11	40.0 VE/mL	16.2 VE/mL
	IgG		41.6 VE/mL	40.2 VE/mL
*Borrelia burgdorferi*	IgM		5.9 BBU/mL	6.7 BBU/mL
	IgG		100.0 BBU/mL	100.0 BBU/mL

Abbreviations: BBU, Biomedica Borrelia Unit; VU, VIROTECH Units.

## Data Availability

The data used in this case report cannot be shared publicly due to patient confidentiality.
